# Mortalidade por Insuficiência Cardíaca com Fração de Ejeção Intermediária

**DOI:** 10.36660/abc.20210050

**Published:** 2022-04-07

**Authors:** Giovanni Possamai Dutra, Bruno Ferraz de Oliveira Gomes, Plínio Resende do Carmo, João Luiz Fernandes Petriz, Emilia Matos Nascimento, Basilio de Bragança Pereira, Gláucia Maria Moraes de Oliveira

**Affiliations:** 1 Universidade Federal do Rio de Janeiro Rio de Janeiro RJ Brasil Universidade Federal do Rio de Janeiro, Rio de Janeiro, RJ – Brasil; 2 Hospital Barra D’or Rio de Janeiro RJ Brasil Hospital Barra D’or – Cardiologia, Rio de Janeiro, RJ – Brasil; 3 UEZO Rio de Janeiro RJ Brasil Centro Universitário Estadual da Zona Oeste – UEZO, Rio de Janeiro, RJ – Brasil

**Keywords:** Insuficiência Cardíaca, Mortalidade, Fração de Ejeção Intermediária

## Abstract

**Fundamento:**

A importância prognóstica da classificação ‘insuficiência cardíaca (IC) com fração de ejeção (FE) intermediária’ permanece incerta.

**Objetivo:**

Analisar as características clínicas, comorbidades, complicações e mortalidade hospitalar e tardia de pacientes classificados em IC com FE intermediária (ICFEi - FE: 40%-49%) e comparar às daqueles em IC com FE preservada (ICFEp - FE > 50%) e IC com FE reduzida (ICFEr - FE < 40%) na internação por IC descompensada.

**Métodos:**

Coorte ambispectiva de pacientes internados por IC descompensada em unidade cardiointensiva. Foram avaliadas características clínicas, comorbidades, complicações e mortalidade hospitalar e tardia. Utilizou-se o software R, com significância de 5%, para a realização dos testes qui-quadrado, análises de variância, multivariada de Cox e curva de sobrevida de Kaplan Meier, além de técnicas de machine learning (Elastic Net, árvore de sobrevida).

**Resultados:**

Foram incluídos 519 indivíduos entre setembro de 2011 e junho de 2019, com média da idade de 74,87±13,56 anos, sendo 57,6% homens. Observou-se frequência de ICFEp, ICFEi e ICFEr de 25,4%, 27% e 47,6%, respectivamente. O infarto prévio foi mais frequente na ICFEi. O tempo médio de seguimento foi 2,94±2,55 anos, sem diferença estatística da mortalidade entre os grupos (53,8%, 52,1% e 57,9%). Na curva de sobrevida, não houve diferença entre os grupos ICFEp e ICFEi, nem entre ICFEp e ICFEr, mas houve entre os grupos ICFEi e ICFEr. Idade maior que 77 anos, IC prévia, história de readmissão, demência e necessidade de vasopressores foram associadas com maior mortalidade tardia na árvore de sobrevida.

**Conclusão:**

A FE não foi selecionada como variável associada a mortalidade nos pacientes com IC descompensada.

## Introdução

A insuficiência cardíaca (IC) é uma complexa síndrome clínica de caráter sistêmico, definida como disfunção cardíaca que ocasiona inadequado suprimento sanguíneo para atender às necessidades metabólicas tissulares.^1^ É a terceira causa de morte cardiovascular nos países desenvolvidos e importante causa de morbidade e hospitalização.^2^ No Brasil, a taxa de mortalidade por IC em números absolutos apresentou um declínio não significativo de 2008 a 2015.^3^ No registro BREATHE, o primeiro registro nacional e multicêntrico de IC aguda do Brasil, pacientes com IC descompensada apresentaram elevada taxa de mortalidade hospitalar.^4^ A IC foi a principal causa cardiovascular de hospitalizações no Brasil entre 2008 e 2017, com 2.380.133 autorizações de internação hospitalar pagas, cerca de 21% do total.^3^

A mortalidade relacionada com IC, bem como a necessidade de internação por essa causa, está intimamente associada com a avaliação da fração de ejeção (FE) do ventrículo esquerdo, que é empregada para diagnóstico, tratamento e prognóstico dessa síndrome. Em 2016, a Sociedade Europeia de Cardiologia publicou uma diretriz com nova proposta de classificação da FE, introduzindo o conceito de IC com FE intermediária (ICFEi) para os pacientes com FE entre 40% e 49%.^5^ Nessa classificação, a IC com FE maior do que 50% foi denominada IC com FE preservada (ICFEp) e, quando a FE foi menor que 40%, IC com FE reduzida (ICFEr).^5^

A relevância para a prática clínica da classificação de ICFEi permanece incerta em relação à mudança de abordagem diagnóstica e terapêutica individualizada para essa categoria. O estudo CHART-2, publicado em 2017, com 3.480 pacientes do Registro do Distrito de Tohoku seguidos por 1 ano, demonstrou que as características clínicas dos pacientes com ICFEi eram distintas, sugerindo que a ICFEi representava uma faixa de transição entre ICFEp e ICFEr ou uma sobreposição do limite inferior da ICFEp com o limite superior da ICFEr.^6^

Devido às dúvidas ainda existentes na literatura, o objetivo deste artigo foi analisar as características clínicas, comorbidades, complicações e mortalidade hospitalar e tardia de pacientes classificados com ICFEi e compará-las às daqueles com ICFEp e ICFEr na internação por IC descompensada. A análise desses dados poderá permitir melhor entendimento sobre a importância da ICFEi para a abordagem terapêutica e o prognóstico de pacientes brasileiros hospitalizados por IC.

## Métodos

Coorte ambispectiva de pacientes internados em unidade cardiointensiva por IC descompensada no período de setembro de 2011 a junho de 2019. Foram incluídos pacientes com idade superior a 18 anos que preencheram os critérios de Framingham e Boston. Foram excluídas 203 internações múltiplas, sendo considerada somente a última internação. A informação sobre mortalidade tardia por todas as causas foi extraída do site da Corregedoria Geral da Justiça do Rio de Janeiro (http://www4.tjrj.jus.br/SEIDEWEB/default.aspx). Os pacientes foram analisados por 3 anos para o desfecho morte por todas as causas.

Foram analisadas as seguintes variáveis: idade, gênero, frequência cardíaca de admissão, história familiar de doença arterial coronariana e revascularização miocárdica e presença de comorbidades, como diabetes, hipertensão, fibrilação atrial, doença renal crônica (taxa de filtração glomerular < 60ml/min/1,73m^2^), infarto, IC, acidente vascular cerebral e demência. Avaliou-se o uso prévio de betabloqueadores, inibidores da enzima de conversão da angiotensina (IECA), bloqueador dos receptores de angiotensina (BRA) e nitratos. Foram avaliados: creatinina e BNP de admissão, necessidade de realização de angiografia coronária, uso de cateter vesical de demora, emprego de vasopressores e tratamento dialítico.

As variáveis foram coletadas em questionário padronizado. Foi utilizado o ecocardiograma da admissão com o método de Teichholz ou Simpson para aferição e classificação da FE. Os pacientes foram separados em três grupos de acordo com a FE, considerando-se ICFEp, ICFEi e ICFEr em conformidade com a classificação proposta pela última diretriz.^5,7^

O projeto foi submetido ao Conselho de Ética em Pesquisa e aprovado conforme parecer emitido em 18/09/2019 sob o Certificado de Apresentação para Apreciação Ética de número 18502319.3.0000.5249 (Parecer: 3.582.453), com dispensa da utilização do termo de consentimento livre e esclarecido, por se tratar de análise ambispectiva de banco de dados coletados de forma parcialmente prospectiva.

### Análise estatística

A normalidade das variáveis contínuas foi avaliada através do teste de Kolmogorov-Smirnov. Os resultados foram exibidos através de média ± desvio padrão (variáveis contínuas) ou número de ocorrência com percentual (variáveis categóricas). Foram utilizados o teste de qui-quadrado para variáveis categóricas e a análise de variância (ANOVA unidirecional) para comparar médias. A curva de Kaplan-Meier foi empregada para analisar a sobrevivência ao longo do tempo, utilizando-se o teste Tarone-Ware para as comparações entre os grupos.^8,9^

Empregou-se o modelo semi-paramétrico de Cox, sequencialmente estimado através de Elastic Net, técnica de regularização em machine learning, para seleção inicial de variáveis, em seguida reestimado através de máxima verossimilhança guardando-se as variáveis significantes. Empregou-se a árvore de sobrevida (machine learning) para identificação das variáveis explicativas da mortalidade ao longo do tempo. O software R foi utilizado para as análises estatísticas com nível de significância de 5%.^10^

As larguras dos intervalos de confiança não foram ajustadas para multiplicidade e, portanto, eles não devem ser usados para inferir o tratamento definitivo. Os modelos de Cox foram usados para calcular as medidas de associação (riscos relativos) e seus respectivos intervalos de confiança de 95%.

## Resultados

Foram incluídos 519 indivíduos com média de idade de 74,87 ± 13,56 anos e maioria de homens (57,6%). As distribuições das frequências de ICFEp, ICFEi e ICFEr foram 25,4%, 27% e 47,6%, respectivamente. Todas as variáveis contínuas exibiram comportamento de normalidade. O sexo masculino foi mais frequente nos grupos com ICFEi e ICFEr comparado ao ICFEp. A ocorrência de IC prévia e fibrilação atrial permanente foi significativamente maior na ICFEp, enquanto a de infarto do miocárdio prévio foi maior na ICFEi. O uso prévio de betabloqueadores foi semelhante entre os grupos, e o de IECA e BRA foi maior nos grupos com ICFEi e ICFEr. Houve um gradiente crescente entre a necessidade do uso de vasopressores e a diminuição da FE (Tabela 1).

**Tabela 1:** Características clínicas dos pacientes portadores de insuficiência cardíaca com fração de ejeção preservada, intermediária e reduzida

Variáveis	ICFEp	ICFEi	ICFEr	Total	p
n (%)	132(25,4%)	140(27%)	247(47,6%)	519	-
Idade (média)	77,8±15,8	74,2±11,9	73,6±12,8	74,8±13,5	0,13^#^
Homens	45(34,1%)	87(62,1%)	167(67,6%)	299(57,6%)	**<0,001**
FE (média)	66,9±8,9	45,1±3,3	30,3±7,6	43,6±16,6	**<0,001** ^#^
BNP (médio)	3807	4969	6301	5307	0,17^#^
DM	43(32,6%)	52(37,1%)	93(37,8%)	188(36,2%)	0,59
HAS	109(82,6%)	109(77,9%)	191(77,3%)	409(78,8%)	0,46
FA permanente	40(30,3%)	20(14,3%)	41(16,6%)	101(19,5%)	**0,001**
DRC* (TFG <60ml/min/1,73m^2^)	21(15,9%)	26(18,6%)	30(12,1%)	77(14,8%)	0,21
IM *	22(16,7%)	48(34,3%)	65(26,3%)	135(26,3%)	**0,004**
IC *	56(42,4%)	35(25%)	96(38,9%)	187(36%)	**0,005**
AVC*	12(9,1%)	9(6,4%)	37(6,5%)	37(7,1%)	0,59
Demência prévia	14(10,6%)	15(10,7%)	17(6,9%)	46(8,9%)	0,32
Betabloqueador prévio	55(41,7%)	60(42,9%)	94(38,1%)	209(40,3%)	0,60
IECA/BRA prévio	13(9,8%)	48(34,3%)	73(29,3%)	134(25,8%)	**<0,001**
Uso de vasopressores	10(7,6%)	21(15%)	59(23,9%)	90(17,3%)	**<0,001**

Valores apresentados como média e desvio padrão. IC: insuficiência cardíaca; FE: fração de ejeção; ICFEp: IC com FE preservada; ICFEi: IC com FE intermediária; ICFEr: IC com FE reduzida; BRA: bloqueador dos receptores de angiotensina; BNP: peptídeo natriurético cerebral; DM: diabetes mellitus; DRC: doença renal crônica; FA: fibrilação atrial; HAS: hipertensão arterial sistêmica; IM: infarto do miocárdio; IECA: inibidores da enzima conversora de angiotensina; AVC: acidente vascular cerebral; TFG: taxa de filtração glomerular. (*) existentes quando da internação; # ANOVA, demais variáveis, qui-quadrado.

**Figura 1:**
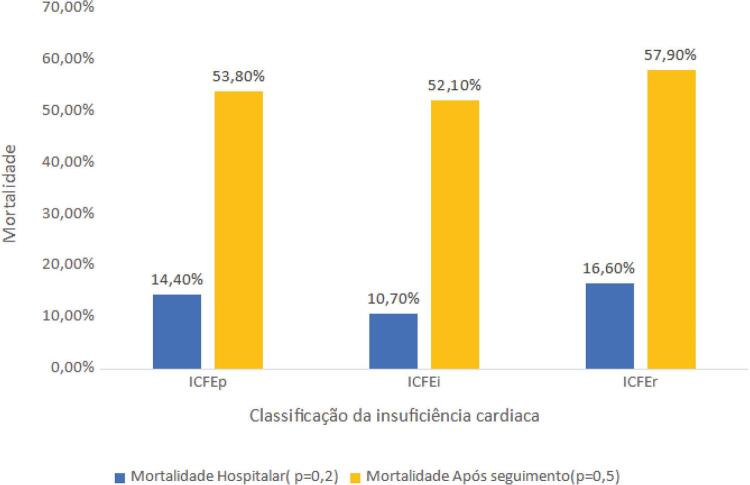
Mortalidade hospitalar e após o seguimento (2,94 anos) em pacientes hospitalizados por insuficiência cardíaca com fração de ejeção preservada (ICFEp), insuficiência cardíaca com fração de ejeção intermediária (ICFEi) e insuficiência cardíaca com fração de ejeção reduzida (ICFEr).

O tempo de seguimento médio foi de 2,94 ± 2,55 anos. No período de seguimento, 287 (52,3%) pacientes foram a óbito e 75 (14,5%) faleceram durante o período de hospitalização, não havendo diferença estatística entre os grupos (Figura 1). Na análise das causas específicas de óbito hospitalar, observou-se maior frequência das causas infecciosas, representadas pela septicemia e pneumonia, com 7,3% e 4,2% do total de menções, respectivamente. Na sequência, vieram as doenças do aparelho circulatório, representadas pela IC e doença isquêmica aguda e crônica do coração, que foram responsáveis por 5,6%, 3,7% e 3,4% do total de menções, respectivamente.

**Figura 2:**
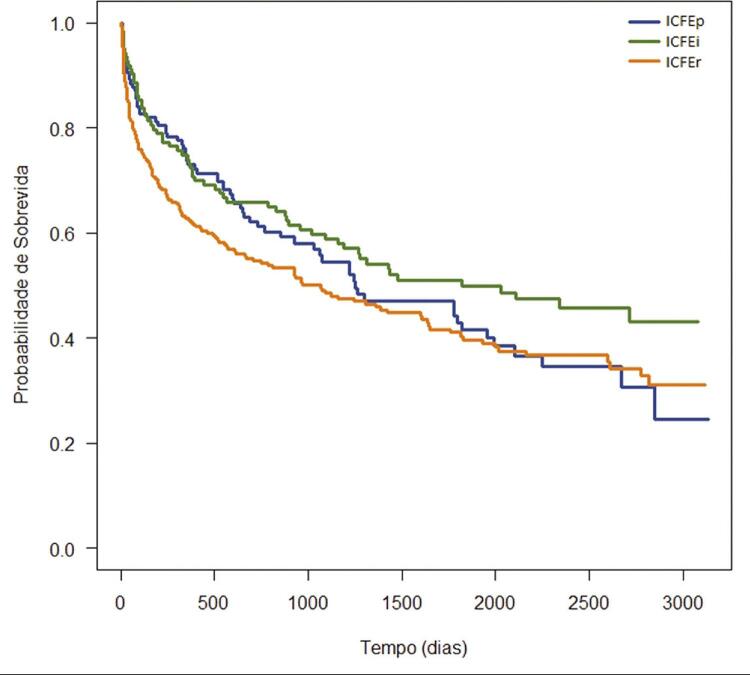
Sobrevida (Curva de Kaplan-Meier8) dos pacientes com insuficiência cardíaca com fração de ejeção preservada (ICFEp), insuficiência cardíaca com fração de ejeção intermediária (ICFEi) e insuficiência cardíaca com fração de ejeção reduzida (ICFEr).

Na curva de sobrevida de Kaplan-Meier^8^ (Figura 2), o teste Tarone Ware^10^ indica que não há diferença significativa na comparação da sobrevida entre os grupos com ICFEp e ICFEi (p=0,27) e entre aqueles com ICFEp e ICFEr (p=0,21). Por outro lado, houve diferença estatística significante entre os grupos com ICFEi e ICFEr (p=0,02).

A análise multivariada do modelo de Cox (Tabela 2) identificou 13 variáveis associadas ao risco de morte no tempo de seguimento. Entre essas variáveis, destacam-se por sua importância clínica e maior risco relativo: necessidade de monitoração do débito urinário com cateter vesical de demora, relato de readmissão, cirurgia de revascularização miocárdica prévia, demência e IC prévias, necessidade de tratamento dialítico e uso de vasopressores.

**Tabela 2:** Modelo de Cox para o desfecho mortalidade com seguimento médio foi de 2,94 ± 2,55 anos

Variáveis	Coeficiente (RR)	Intervalo de confiança 95%	p valor
HFDAC	0,56	0,33 -0,96	0,037
Realização de angiografia coronariana	0,61	0,38 - 0,99	0,004
Nitrato prévio	0,68	0,51 - 0,91	0,009
Creatinina de admissão	0,88	0,79 - 0.98	0,002
FC de admissão	0,98	0,98 - 0,99	0,001
Idade	1,03	1,02 – 1,04	<0,001
Uso de CVD	1,48	1,14 - 1,94	<0,001
Readmissão	1,52	1,18 – 1,96	0,001
CRVM prévia	1,63	1,13 - 2,35	0,008
Demência	1,72	1,21 - 2.44	0,002
IC prévia	2,24	1,73 - 2,90	<0,001
Tratamento dialítico	2,56	1,62 - 4,04	<0,001
Vasopressor	2,91	2,06 - 4,11	<0,001

*RR: risco relativo; HFDAC: história familiar de doença arterial coronariana; FC: frequência cardíaca; CVD: cateter vesical de demora; CRVM: cirurgia de revascularização miocárdica; IC: insuficiência cardíaca.*

**Figura 3:**
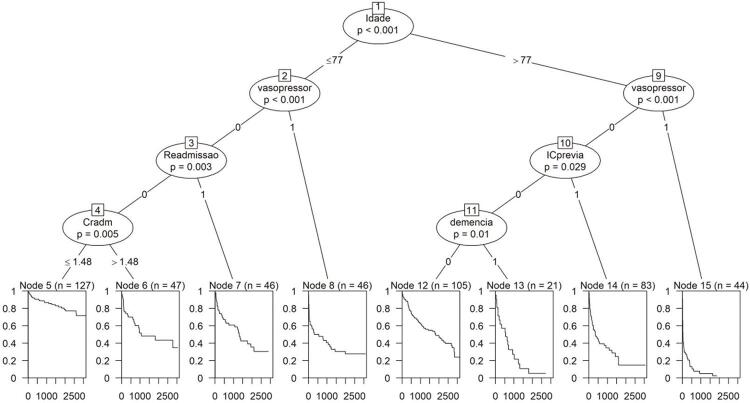
Árvore de sobrevivência dos pacientes internados com insuficiência cardíaca. Cradm: creatinina na admissão; IC: insuficiência cardíaca.

A árvore de sobrevivência auxilia na identificação de padrões de menor sobrevida, considerando todas as variáveis em conjunto (Figura 3). A idade acima de 77 anos e a necessidade de vasopressores foram associadas com maior mortalidade. O segundo padrão de maior mortalidade foi dos pacientes com mais de 77 anos que tinham IC prévia ou demência. O uso de vasopressor e a readmissão foram o terceiro padrão associado a maior mortalidade independentemente da idade. A creatinina de admissão maior que 1,48 mg/dl foi o padrão subsequente de maior mortalidade.

## Discussão

Este artigo avaliou uma coorte prospectiva de indivíduos internados por IC descompensada e empregou inteligência artificial para identificar características próprias da ICFEi quanto a mortalidade hospitalar e tardia, relacionando-a com os grupos categorizados conforme a FE. O infarto prévio foi mais frequente na ICFEi e não houve diferença estatística de mortalidade entre os grupos no seguimento de 2,94 ± 2,55 anos. Na curva de sobrevida, também não houve diferença entre os pacientes com ICFEp e ICFEi, nem entre aqueles com ICFEp e ICFEr, mas observou-se diferença estatística entre pacientes com ICFEi e ICFEr. Idade maior que 77 anos, presença de IC prévia, história de readmissão, presença de demência e necessidade de vasopressores foram associadas com maior mortalidade tardia na árvore de sobrevida. Cabe ressaltar que a FE não foi selecionada como variável associada com mortalidade nos pacientes com IC descompensada.

Metanálise publicada em 2018, com 606.762 pacientes adultos, comparou a taxa de hospitalização e a mortalidade da ICFEi com as da ICFEp e ICFEr. Os resultados sugeriram diferenças significativas na mortalidade por todas as causas e na mortalidade não cardíaca entre os pacientes com ICFEr e ICFEi. Por outro lado, a ICFEp diferiu significativamente da ICFEi em relação à morte cardíaca. A hospitalização relacionada a IC não apresentou distinção entre os grupos.^11^ Esse achado foi semelhante ao observado no presente estudo, onde a mortalidade por todas as causas foi diferente entre os grupos da ICFEi e ICFEr. Os autores salientaram a importância das comorbidades concomitantes para os achados relacionados com a mortalidade.^11^ Além disso, observamos maior prevalência de infarto do miocárdio nos pacientes com ICFEi, assim como de fibrilação atrial permanente no grupo da ICFEp.

Outra metanálise de 2018, com 109.257 pacientes oriundos de 12 estudos, analisou as características clínicas, a hospitalização e a mortalidade por todas as causas nos três grupos categorizados de acordo com a FE. Os autores observaram diferenças marcantes nas características basais, na mortalidade cardiovascular e por todas as causas e na internação por IC entre as três categorias. Nessa metanálise, os pacientes com ICFEi eram idosos, com predominância de homens, apresentando menos doença isquêmica do coração em comparação aos pacientes com ICFEr.^12^ Observou-se um gradiente de frequência entre idade, gênero, presença de doença isquêmica do coração, hipertensão, fibrilação atrial, doença pulmonar obstrutiva crônica e redução da taxa de filtração glomerular em relação à categorização da FE. O mesmo ocorreu com o uso de betabloqueadores e IECA. Em aproximadamente 3 anos, as mortes por todas as causas foram em número menor na ICFEi do que na ICFEr, porém maior do que na ICFEp. De forma similar, a mortalidade de causa cardiovascular e as hospitalizações foram menores na ICFEi quando comparada à ICFEr e discretamente maior quando comparada à ICFEp. Esses achados também sugerem que a ICFEi, considerando os dados e desfechos, ocupa posição intermediária, sendo mais associada a desfechos de pior prognóstico que a ICFEp, porém menos associada que a ICFEr. Uma observação relevante é que as populações dos estudos incluídos eram heterogêneas e observacionais, com amostras de diferentes tamanhos. Somente cinco dos estudos tinham dados de hospitalização por IC e morte cardiovascular, demonstrando que o resultado deve ser interpretado de forma cautelosa.^12^

Cabe salientar que os estudos citados não consideraram a relação das variáveis e suas associações com os desfechos ao longo do tempo, bem como as interações entre todas as variáveis. A média da idade do nosso estudo foi de aproximadamente 75 anos, pouco elevada em comparação com a literatura, com a metanálise anterior (62 anos) ou o registro BREATHE (64 anos).^4^ Essa pode ser uma das razões do ponto de corte de 77 anos na árvore de sobrevida. Também houve predominância do sexo masculino entre os pacientes com ICFEi e ICFEr.^13^

As causas infecciosas, septicemia e pneumonia, foram listadas como as maiores mortalidades específicas hospitalares nessa amostra. Estudo^14^ demonstrou que o prognóstico cardiovascular de IC de início recente melhorou substancialmente entre 2002 e 2014 (razão de risco: 0,73; IC95%: 0,68-0,80) para pacientes acima e abaixo da idade de 80 anos. No entanto, naqueles com idade maior que 80 anos, a queda na mortalidade cardiovascular foi totalmente compensada pela mortalidade por causa não cardiovascular. O tratamento, nesse caso, mudou a forma como os pacientes idosos morreram, como observado em nosso estudo.

A presença de síndrome demencial, especialmente não relacionada ao uso de vasopressores na internação, foi um fator de pior prognóstico demonstrado na análise de sobrevivência. Estudo recente relatou declínio funcional em 15% dos pacientes, sendo, em 80% desses, prévio à internação por IC descompensada, que foi associado a maior risco de longo prazo para desfecho composto por hospitalização e morte por todas as causas ou IC, semelhante aos nossos achados.^15^

A presença de IC prévia nesta amostra foi relacionada à maior mortalidade ao longo do tempo, bem como às readmissões por IC e à necessidade de agentes inotrópicos, identificados pela técnica de *machine learning*. Essas três variáveis indicam piora de prognóstico dos pacientes internados por IC descompensada no longo prazo, sendo marcadores de gravidade que independem da FE. Pacientes internados por IC têm elevada taxa de re-hospitalização em até 6 meses (30% a 40%)^16^ e o risco de morte após hospitalização por IC permanece aumentado entre 12 e 18 meses após o evento índice,^17^ sendo uma das variáveis utilizadas para indicação de transplante cardíaco.^18^ As taxas de readmissão por IC em adultos jovens são similares às de idosos, o que sugere que o risco de re-hospitalização está presente independentemente da idade.^19^

Disfunção renal crônica e IC frequentemente coexistem, compartilhando muitos fatores de risco, como diabetes, hipertensão e hiperlipidemia, o que agrava o prognóstico da IC crônica descompensada.^20^ A síndrome cardiorrenal, caracterizada por agravamento da função renal durante a hospitalização por IC ou logo após a alta, também concorre para piorar o prognóstico da IC descompensada.^21^ A creatinina de admissão maior que 1,48 mg/dl foi associada a pior prognóstico nos indivíduos com idade inferior a 77 anos, representando maior risco para disfunção renal, síndrome cardiorrenal e necessidade de terapia dialítica.

Existem vários modelos de previsão de mortalidade para IC, como o *Get With the Guidelines-Heart Failure* (GWTG-HF)^22^ e o *Meta-Analysis Global Group in Chronic Heart Failure* (MAGGIC),^23^ com acurácia insatisfatória e sem validação para a população brasileira. Algoritmos que empregam deep learning, como o DAHF, apresentaram melhora da capacidade de predição da mortalidade por IC hospitalar e aos 12 e 36 meses após a internação, mas também não foram desenvolvidos para a população brasileira.^24^ A força deste estudo reside na seleção, por Elastic Net e árvores de sobrevida (machine learning), de padrões de apresentação clínica associados com pior mortalidade hospitalar e tardia de pacientes internados com IC descompensada em unidade cardiointensiva brasileira.

Este estudo tem como limitação sua realização em um único centro e a ausência de coleta de todas as medicações prévias à internação, como o uso de diuréticos. Há, portanto, potencial viés de seleção inerente aos estudos observacionais. Há, nas múltiplas análises entre variáveis independentes e mortalidade, natureza exploratória. Tais características podem comprometer a validade externa dos achados. Sobre a validade interna dos dados, as estatísticas pontuais, como médias e riscos relativos, são mais importantes. A hipótese de que a categorização da FE seria variável preditora de morte hospitalar e tardia nesta amostra não foi corroborada pela análise empregando *machine learning*. Nesse contexto, a morte relacionada a IC descompensada parece representar a soma do envelhecimento às falências orgânicas evolutivas.

## Conclusão

Não houve diferença estatística da mortalidade entre os grupos no seguimento de 2,94 ± 2,55 anos. Na curva de sobrevida, também não houve diferença entre ICFEp e ICFEi, nem entre ICFEp e ICFEr, sendo observada diferença entre pacientes com ICFEi e ICFEr. A idade maior que 77 anos, IC anterior à internação, história de readmissão, presença de demência, creatinina de admissão maior que 1,48 mg/dl e necessidade de vasopressores foram associadas com maior mortalidade tardia na árvore de sobrevida.

## References

[1] Bocchi EA, Marcondes-Braga FG, Bacal F, Ferraz AS, Albuquerque D, Rodrigues DA, et al. Atualização da Diretriz Brasileira de Insuficiência Cardíaca Crônica - 2012. Arq Bras Cardiol. 2012;98(1):1-33. doi: 10.1590/s0066-782x2012001000001. 10.1590/s0066-782x2012001000001 22392082

[2] Martínez RB, Isla JA, Albero MJM. Mortalidad por Insuficiencia Cardíaca en España, 1977-1998. Rev Esp Cardiol. 2002;55(3):219-26. doi: 10.1016/s0300-8932(02)76589-5. 10.1016/s0300-8932(02)76589-5 11893312

[3] Fernandes ADF, Fernandes GC, Mazza MR, Knijnik LM, Fernandes GS, Vilela AT, et al. A 10-Year Trend Analysis of Heart Failure in the Less Developed Brazil. Arq Bras Cardiol. 2020;114(2):222-31. doi: 10.36660/abc.20180321. 10.36660/abc.20180321 PMC7077585 32215488

[4] Albuquerque DCD, Souza JDD, Bacal F, Rohde LEP, Bernardez-Pereira S, Berwanger O, et al . I Registro Brasileiro de Insuficiência Cardíaca – Aspectos Clínicos, Qualidade Assistencial e Desfechos Hospitalares. Arq. Bras. Cardiol. 2015;104(6):433-42. doi: 10.5935/abc.20150031.

[5] Ponikowski P, Voors AA, Anker SD, Bueno H, Cleland JGF, Coats AJS, et al. 2016 ESC Guidelines for the Diagnosis and Treatment of Acute and Chronic Heart Failure: The Task Force for the Diagnosis and Treatment of Acute and Chronic Heart Failure of the European Society of Cardiology (ESC). Eur Heart J. 2016;37(27):2129-200. doi: 10.1093/eurheartj/ehw128. 10.1093/eurheartj/ehw128 27206819

[6] Tsuji K, Sakata Y, Nochioka K, Miura M, Yamauchi T, Onose T, et al. Characterization of Heart Failure Patients with Mid-Range Left Ventricular Ejection Fraction-a Report from the CHART-2 Study. Eur J Heart Fail. 2017;19(10):1258-69. doi: 10.1002/ejhf.807. 10.1002/ejhf.807 28370829

[7] Rohde LEP, Montera MW, Bocchi EA, Clausell NO, Albuquerque DC, Rassi S, et al. Diretriz Brasileira de Insuficiência Cardíaca Crônica e Aguda. Arq Bras Cardiol. 2018;111(3):436-539. doi: 10.5935/abc.20180190. 10.5935/abc.20180190 30379264

[8] Rupert G, Miller JR. Survival Analysis. Hoboken: John Wiley & Sons; 1997.

[9] Tarone RE, Ware J. On Distribution-Free Tests for Equality of Survival Distribution. 1977. Biometrika. 64(1):156-60. doi: 10.2307/2335790.

[10] R Foundation. R: A Language and Environment for Statistical Computing. Vienna: R Foundation for Statistical Computing; 2019.

[11] Altaie S, Khalife W. The Prognosis of Mid-Range Ejection Fraction Heart Failure: A Systematic Review and Meta-Analysis. ESC Heart Fail. 2018;5(6):1008-16. doi: 10.1002/ehf2.12353. 10.1002/ehf2.12353 PMC6301154 30211480

[12] Lauritsen J, Gustafsson F, Abdulla J. Characteristics and Long-Term Prognosis of Patients with Heart Failure and Mid-Range Ejection Fraction Compared with Reduced and Preserved Ejection Fraction: A Systematic Review and Meta-Analysis. ESC Heart Fail. 2018;5(4):685-94. doi: 10.1002/ehf2.12283. 10.1002/ehf2.12283 PMC6073025 29660263

[13] Hsich EM, Grau-Sepulveda MV, Hernandez AF, Eapen ZJ, Xian Y, Schwamm LH, Bhatt DL, Fonarow GC. Relationship Between Sex, Ejection Fraction, and B-type Natriuretic Peptide Levels in Patients Hospitalized with Heart Failure and Associations with Inhospital Outcomes: Findings from the Get with The Guideline-Heart Failure Registry. Am Heart J. 2013;166(6):1063-1071.e3. doi: 10.1016/j.ahj.2013.08.029. 10.1016/j.ahj.2013.08.029 24268222

[14] Conrad N, Judge A, Canoy D, Tran J, Pinho-Gomes AC, Millett ERC, et al. Temporal Trends and Patterns in Mortality After Incident Heart Failure: A Longitudinal Analysis of 86 000 Individuals. JAMA Cardiol. 2019;4(11):1102-111. doi: 10.1001/jamacardio.2019.3593. 10.1001/jamacardio.2019.3593 PMC6724155 31479100

[15] Yaku H, Kato T, Morimoto T, Inuzuka Y, Tamaki Y, Ozasa N, et al. Risk Factors and Clinical Outcomes of Functional Decline During Hospitalisation in Very Old Patients with Acute Decompensated Heart Failure: An Observational Study. BMJ Open. 2020;10(2):e032674. doi: 10.1136/bmjopen-2019-032674. 10.1136/bmjopen-2019-032674 PMC7044905 32066601

[16] Wong CY, Chaudhry SI, Desai MM, Krumholz HM. Trends in Comorbidity, Disability, and Polypharmacy in Heart Failure. Am J Med. 2011;124(2):136-43. doi: 10.1016/j.amjmed.2010.08.017. 10.1016/j.amjmed.2010.08.017 PMC3237399 21295193

[17] Kristensen SL, Jhund PS, Køber L, Preiss D, Kjekshus J, McKelvie RS, et al. Comparison of Outcomes After Hospitalization for Worsening Heart Failure, Myocardial Infarction, and Stroke in Patients with Heart Failure and Reduced and Preserved Ejection Fraction. Eur J Heart Fail. 2015;17(2):169-76. doi: 10.1002/ejhf.211. 10.1002/ejhf.211 25756844

[18] Bacal F, Marcondes-Braga FG, Rohde LEP, Xavier Júnior JL, Brito FS, Moura LAZ, et al. 3ª Diretriz Brasileira de Transplante Cardíaco. Arq. Bras. Cardiol. 2018;111(2):230-89. 10.5935/abc.20180153 30335870

[19] Ranasinghe I, Wang Y, Dharmarajan K, Hsieh AF, Bernheim SM, Krumholz HM. Readmissions After Hospitalization for Heart Failure, Acute Myocardial Infarction, or Pneumonia Among Young and Middle-Aged Adults: A Retrospective Observational Cohort Study. PLoS Med. 2014;11(9):e1001737. doi: 10.1371/journal.pmed.1001737. 10.1371/journal.pmed.1001737 PMC4181962 25268126

[20] Damman K, Valente MA, Voors AA, O’Connor CM, van Veldhuisen DJ, Hillege HL. Renal Impairment, Worsening Renal Function, and Outcome in Patients with Heart Failure: An Updated Meta-Analysis. Eur Heart J. 2014;35(7):455-69. doi: 10.1093/eurheartj/eht386. 10.1093/eurheartj/eht386 24164864

[21] Bock JS, Gottlieb SS. Cardiorenal Syndrome: New Perspectives. Circulation. 2010;121(23):2592-600. doi: 10.1161/CIRCULATIONAHA.109.886473. 10.1161/CIRCULATIONAHA.109.886473 20547939

[22] Lagu T, Pekow PS, Shieh MS, Stefan M, Pack QR, Kashef MA, et al. Validation and Comparison of Seven Mortality Prediction Models for Hospitalized Patients with Acute Decompensated Heart Failure. Circ Heart Fail. 2016;9(8). doi: 10.1161/CIRCHEARTFAILURE.115.002912. 10.1161/CIRCHEARTFAILURE.115.002912 PMC4988343 27514749

[23] Pocock SJ, Ariti CA, McMurray JJ, Maggioni A, Køber L, Squire IB, et al. Predicting Survival in Heart Failure: A Risk Score Based on 39 372 Patients from 30 Studies. Eur Heart J. 2013;34(19):1404-13. doi: 10.1093/eurheartj/ehs337. 10.1093/eurheartj/ehs337 23095984

[24] Kwon JM, Kim KH, Jeon KH, Lee SE, Lee HY, Cho HJ, et al. Artificial Intelligence Algorithm for Predicting Mortality of Patients with Acute Heart Failure. PLoS One. 2019;14(7):e0219302. doi: 10.1371/journal.pone.0219302. 10.1371/journal.pone.0219302 PMC6613702 31283783

