# Supplementary material for: Mortalidade por Insuficiência Cardíaca com Fração de Ejeção Intermediária
Source: Arq Bras Cardiol. 2022 Apr 7;118(4):694–700. [Article in Portuguese] doi: 10.36660/abc.20210050 (PMC9007002; doi:10.36660/abc.20210050)
Supplement: Translation

# Translation: Mortality from Heart Failure with Mid-Range Ejection Fraction

**Authors:** Giovanni Possamai Dutra, Bruno Ferraz de Oliveira Gomes, Plínio Resende do Carmo, João Luiz Fernandes Petriz, Emilia Matos Nascimento, Basilio de Bragança Pereira, Gláucia Maria Moraes de Oliveira

**Affiliations:** 1 Brazil Universidade Federal do Rio de Janeiro, Rio de Janeiro, RJ – Brazil; 2 Brazil Hospital Barra D’or – Cardiologia, Rio de Janeiro, RJ – Brazil; 3 Brazil Centro Universitário Estadual da Zona Oeste – UEZO, Rio de Janeiro, RJ – Brazil

**Keywords:** Heart Failure, Mortality, Mid-Range Ejection Fraction

## Abstract

**Background:**

The prognostic importance of the classification ‘heart failure (HF) with mid-range ejection fraction (EF)’ remains uncertain.

**Objective:**

To analyze the clinical characteristics, comorbidities, complications, and in-hospital and late mortality of patients classified as having HF with mid-range EF (HFmrEF – EF: 40%-49%), and to compare them to those of patients with HF with preserved EF (HFpEF – EF > 50%) and with HF with reduced EF (HFrEF – EF < 40%) on admission for decompensated HF.

**Methods:**

Ambispective cohort of patients admitted to the cardiac intensive care unit due to decompensated HF. Clinical characteristics, comorbidities, complications, and in-hospital and late mortality were assessed. The software R was used, with a 5% significance, for the tests chi-square, analysis of variance, Cox multivariate, and Kaplan-Meier survival curve, in addition to machine-learning techniques (Elastic Net and survival tree).

**Results:**

519 individuals were included between September 2011 and June 2019 (mean age, 74.87 ± 13.56 years; 57.6% were men). The frequencies of HFpEF, HFmrEF and HFrEF were 25.4%, 27% and 47.6%, respectively. Previous infarction was more frequent in HFmrEF. The mean follow-up time was 2.94 ± 2.55 years, with no statistical difference in mortality between the groups (53.8%, 52.1%, 57.9%). In the survival curve, there was difference between neither the HFpEF and HFmrEF groups, nor the HFpEF and HFrEF groups, but between the HFmrEF and HFrEF groups. Age over 77 years, previous HF, history of readmission, dementia and need for vasopressors were associated with higher late mortality in the survival tree.

**Conclusion:**

The EF was not selected as a variable associated with mortality in patients with decompensated HF.

## Introduction

Heart failure (HF) is a complex systemic clinical syndrome, defined as cardiac dysfunction that causes inadequate blood supply to meet tissue metabolic needs.^1^ It is the third cause of cardiovascular death in developed countries and an important cause of morbidity and hospitalization.^2^ In Brazil, the mortality rate from HF in absolute numbers had a non significant decrease from 2008 to 2015.^3^ In the BREATHE registry, the first Brazilian multicenter registry of acute HF, patients with decompensated HF had a high in-hospital mortality rate.^4^ Heart failure was the main cardiovascular cause of hospitalizations in Brazil between 2008 and 2017, with 2.380.133 paid authorizations for hospitalization, accounting for 21% of the total number.^3^

Mortality related to HF, as well as the need for admission due to that syndrome, is closely associated with the assessment of left ventricular ejection fraction (EF), which is used for HF diagnosis, treatment, and prognosis. In 2016, The European Society of Cardiology issued a HF guideline with a new EF classification, introducing the concept of HF with mid-range EF (HFmrEF) for patients with EF ranging from 40% to 49%.^5^ According to that classification, HF with EF equal to or greater than 50% was named HF with preserved EF (HFpEF), while HF with EF below 40% was named HF with reduced EF (HFrEF).^5^

The relevance of the HFmrEF classification for clinical practice remains uncertain regarding the change in the individualized diagnostic and therapeutic approach for that category. The CHART-2 Study, published in 2017, with 3480 patients from the ‘Registry in the Tohoku District’ followed up for 1 year, has shown that the clinical characteristics of patients with HFmrEF were different, suggesting that HFmrEF represented a transitional status or an overlap zone between HFpEF and HFrEF.^6^

Because of the remaining doubts in the literature, this study aimed to analyze the clinical characteristics, comorbidities, complications, and in-hospital and late mortality of patients classified as having HFmrEF, in addition to compare them to those of patients with HFpEF and HFrEF on admission due to decompensated HF. The analysis of those data can provide better understanding about the importance of HFmrEF for the therapeutic approach and prognosis of Brazilian patients admitted due to HF.

## Methods

Ambispective cohort of patients admitted to the cardiac intensive care unit due to decompensated HF, from September 2011 to June 2019. Patients aged > 18 years and meeting the Framingham and Boston criteria were included, while 203 multiple admissions were excluded, only the last admission being considered. Information on late all-cause mortality was extracted from the site of the General Internal Affairs of Justice from Rio de Janeiro (http://www4.tjrj.jus.br/SEIDEWEB/default.aspx). Patients were assessed for 3 years regarding the outcome ‘death from all causes’.

The following variables were assessed: age, sex, heart rate on admission, family history of coronary artery disease and myocardial revascularization, and presence of comorbidities, such as diabetes, hypertension, atrial fibrillation, chronic kidney disease (glomerular filtration rate < 60mL/min/1.73m^2^), infarction, HF, stroke, and dementia. The previous use of beta-blockers, angiotensin-converting-enzyme inhibitor (ACEI), angiotensin receptor blocker (ARB), and nitrates was evaluated. In addition, the following were assessed: creatinine and BNP levels on admission; need for coronary angiography; use of indwelling urinary catheter and of vasopressors; and dialysis treatment.

The variables were collected by using a standardized questionnaire. Echocardiogram on admission and the Teichholz’s formula or Simpson’s rule were used to measured and classify EF. The patients were separated into three groups according to their EF, considering HFpEF, HFmrEF and HFrEF in accordance with the classification of the last guideline.^5,7^

This study’s project was submitted to and approved by the Committee on Ethics and Research on 09/18/2019 (certification of presentation for ethical appreciation number 18502319.3.0000.5249; appraisal: 3.582.453). Because this is an ambispective analysis of data collected in a partially prospective way, written informed consent was waived.

### Statistical analysis

The normal distribution of the continuous variables was assessed by use of the Kolmogorov-Smirnov test. The results were presented as mean ± standard deviation (continuous variables) or number of occurrence and percentage (categorical variables). The means were compared by use of the chi-square test for categorical variables and analysis of variance (1-way ANOVA). The Kaplan-Meier curve was used to analyze survival over time, and the Tarone-Ware test for comparisons between the groups.^8,9^

The semi-parametric Cox model, sequentially estimated by use of Elastic Net, a machine-learning regularization technique, was used for the initial selection of variables, and then re-estimated by use of maximum likelihood and the significant variables put aside. Survival tree (machine learning) was used to identify the explanatory variables of mortality over time. The software R was used for statistical analyses at 5% significance level.^10^

The widths of the confidence intervals were not adjusted to multiplicity, thus, they should noy be used to infer the definitive treatment. The Cox models were used to calculate the measures of association (relative risks) and their respective 95% confidence intervals.

## Results

This study included 519 individuals with a mean age of 74.87 ± 13.56 years, and 57.6% were men. The frequency distributions of HFpEF, HFmrEF and HFrEF were 25.4%, 27%, and 47.6%, respectively. All continuous variables were normally distributed. The male sex was more frequent in the HFmrEF and HFrEF groups as compared to the HFpEF group. The occurrence of previous HF and permanent atrial fibrillation was significantly higher in the HFpEF group, while that of previous myocardial infarction was higher in the HFmrEF group. The previous use of beta-blockers was similar in the groups, while that of ACEI and ARB was higher in the HFmrEF and HFrEF groups. There was an increasing gradient between the need for vasopressor use and the EF reduction (Table 1).

**Table 1:** Clinical characteristics of patients with heart failure with preserved, mid-range and reduced ejection fraction

Variables	HFpEF	HFmrEF	HFrEF	Total	p
n (%)	132(25.4%)	140(27%)	247(47.6%)	519	-
Age (mean)	77.8±15.8	74.2±11.9	73.6±12.8	74.8±13.5	0.13^#^
Men	45(34.1%)	87(62.1%)	167(67.6%)	299(57.6%)	**<0.001**
EF (mean)	66.9±8.9	45.1±3.3	30.3±7.6	43.6±16.6	**<0.001** ^#^
BNP (mean)	3807	4969	6301	5307	0.17^#^
DM	43(32.6%)	52(37.1%)	93(37.8%)	188(36.2%)	0.59
SAH	109(82.6%)	109(77.9%)	91(77.3%)	409(78.8%)	0.46
Permanent AF	40(30.3%)	20(14.3%)	41(16.6%)	101(19.5%)	**0.001**
CKD* (GFR <60ml/min/1.73m2)	21(15.9%)	26(18.6%)	30(12.1%)	77(14.8%)	0.21
MI *	22(16.7%)	48(34.3%)	65(26.3%)	135(26.3%)	**0.004**
HF *	56(42.4%)	35(25%)	96(38.9%)	187(36%)	**0.005**
Stroke*	12(9.1%)	9(6.4%)	37(6.5%)	37(7.1%)	0.59
Previous dementia	14(10.6%)	15(10.7%)	17(6.9%)	46(8.9%)	0.32
Previous beta-blocker	55(41.7%)	60(42.9%)	94(38.1%)	209(40.3%)	0.60
Previous ACEI/ARB	13(9.8%)	48(34.3%)	73(29.3%)	134(25.8%)	**<0.001**
Use of vasopressors	10(7.6%)	21(15%)	59(23.9%)	90(17.3%)	**<0.001**

Values shown as mean and standard deviation. HF: heart failure; EF: ejection fraction; HFpEF: HF with preserved EF; HFmrEF: HF with mid-range EF; HFrEF: HF with reduced EF; ACEI: angiotensin-converting-enzyme inhibitor; AF: atrial fibrillation; ARB: angiotensin receptor blocker; BNP: brain natriuretic peptide; CKD: chronic kidney disease; DM: diabetes mellitus; GFR; glomerular filtration rate; MI: myocardial infarction; SAH: systemic arterial hypertension. (*) on admission; # ANOVA, other variables, chi-square.

The mean follow-up duration was 2.94 ± 2.55 years. During follow-up, 287 (52.3%) patients died and, during hospitalization, 75 (14.5%) died, with no statistical difference between groups (Figure 1). When analyzing the specific causes of in-hospital death, there was a higher frequency of infectious causes, represented by septicemia and pneumonia, accounting for 7.3% and 4.2%, respectively. They were followed by diseases of the circulatory system, represented by HF and acute and chronic ischemic heart disease, accounting for 5.6%, 3.7% and 3.4%, respectively.

**Figure 1:** In-hospital mortality e após o follow-up (2,94 years) em patients hospitalizados por HFpEF, HFmrEF e HFrEF.

In the Kaplan-Meier survival curve^8^ (Figure 2), the Tarone Ware test^10^ shows no significant difference when comparing survival between the HFpEF and HFmrEF groups (p=0.27) and between the HFpEF and HFrEF groups (p=0.21). However, there was a significant statistical difference between the HFmrEF and HFrEF groups (p=0.02).

**Figure 2:** Kaplan-Meier survival curve8 of patients with heart failure with preserved ejection fraction (HFpEF), heart failure with mid-range ejection fraction (HFmrEF) and heart failure with reduced ejection fraction (HFrEF) during the study period.

The multivariate analysis of the Cox model (Table 2) identified 13 variables associated with the risk of death during follow-up. Of those variables, the following stand out because of their clinical importance and higher relative risk: need for monitoring of urinary output with indwelling urinary catheter, report of readmission, previous coronary artery bypass grafting surgery, previous dementia and HF, need for dialysis treatment, and use of vasopressors.

**Table 2:** Cox model for the outcome mortality with mean follow-up of 2.94 ± 2.55 years

Variables	Coefficient (RR)	95% confidence interval	p value
FHCAD	0.56	0.33 -0.96	0.037
Coronary angiography	0.61	0.38 - 0.99	0.004
Previous nitrate	0.68	0.51 - 0.91	0.009
Creatinine on admission	0.88	0.79 - 0.98	0.002
HR on admission	0.98	0.98 - 0.99	0.001
Age	1.03	1.02 – 1.04	<0.001
Use of IUC	1.48	1.14 - 1.94	<0.001
Readmission	1.52	1.18 – 1.96	0.001
Previous CABG	1.63	1.13 - 2.35	0.008
Dementia	1.72	1.21 - 2.44	0.002
Previous HF	2.24	1.73 - 2.90	<0.001
Dialysis treatment	2.56	1.62 - 4.04	<0.001
Vasopressor	2.91	2.06 - 4.11	<0.001

*RR: relative risk; FHCAD: family history of coronary artery disease; HR: heart rate; IUC: indwelling urinary catheter; CABG: coronary artery bypass grafting surgery; HF: heart failure.*

The survival tree helps identify the patterns of shorter survival, considering the set of all variables (Figure 3). Age over 77 years and need for vasopressors were associated with higher mortality. The second pattern of higher mortality was patients older than 77 years with previous HF or dementia. The use of vasopressors and readmission were the third pattern associated with higher mortality regardless of age. Creatinine on admission over 1.48 mg/dL was the subsequent pattern of higher mortality.

**Figure 3:** Survival tree of patients admitted due to heart failure. Cradm: creatinine on admission; HF: heart failure.

## Discussion

This study assessed a prospective cohort of patients admitted due to decompensated HF and used artificial intelligence to identify characteristics of HFmrEF regarding in-hospital and late mortality, relating it to the other groups categorized according to EF. Previous infarction was more frequent in HFmrEF and there was no statistical difference in mortality in the groups during the follow-up of 2.94 ± 2.55 years. In addition, in the survival curve, patients with HFpEF did not differ from those with HFmrEF, and patients with HFpEF did not differ from those with HFrEF; however, statistical difference was evidenced between patients with HFmrEF and HFrEF. Age over 77 years, presence of previous HF, history of readmission, presence of dementia and need for vasopressors were associated with higher late mortality in the survival tree. It is worth noting that EF was not selected as a variable associated with mortality in patients with decompensated HF.

Meta-analysis published in 2018, with 606 762 adult patients, compared the hospitalization rate and mortality from HFmrEF to those from HFpEF and HFrEF. The results suggested significant differences in all-cause mortality and noncardiac mortality between the HFrEF and HFmrEF group. In addition, the HFpEF group differed significantly from the HFmrEF group regarding cardiac death. Hospitalization associated with HF showed no difference between the groups.^11^ This finding was similar to that from the present study, in which all-cause mortality differed between the HFmrEF and HFrEF groups. The authors from the meta-analysis emphasized the importance of concomitant comorbidities for the findings related to mortality.^11^ In addition, higher prevalence of myocardial infarction was observed in the HFmrEF group, as well as of permanent atrial fibrillation in the HFpEF group.

Another meta-analysis from 2018 with 109 257 patients from 12 studies analyzed the clinical characteristics, hospitalization, and all-cause mortality in the three groups categorized according to EF. The authors reported significant differences in the baseline characteristics, in cardiovascular and all-cause mortality, and on admission due to HF in the three categories. In that meta-analysis, the patients with HFmrEF were older, mostly men and had less ischemic heart disease as compared to the patients with HFrEF.^12^ A gradient of frequency was observed in age, sex, presence of ischemic heart disease, hypertension, atrial fibrillation, chronic obstructive pulmonary disease, and glomerular filtration rate reduction according to the EF categorization. The same occurred regarding the use of beta-blockers and ACEI. Over approximately 3 years, the number of deaths from all causes was lower in HFmrEF than in HFrEF, but higher than that in HFpEF. Similarly, in HFmrEF, cardiovascular mortality and hospitalizations were lower as compared to HFrEF and slightly higher as compared to HFpEF. These findings suggest that HFmrEF, regarding data and outcomes, occupies a mid-position between HFrEF and HFpEF, more associated with worse prognosis outcomes as compared to HFpEF, but less associated with worse prognosis outcomes as compared to HFrEF. It is worth noting that the studies included were observational with heterogeneous populations and samples of different sizes. Only five studies reported data on hospitalization due to HF and cardiovascular death, indicating that the result should be interpreted carefuly.^12^

It is worth noting that the studies cited considered neither the relationship of the variables and their associations with the outcome over time, nor the interactions between all variables. In our study, the mean age was approximately 75 years, higher than that in the literature, in the cited meta-analysis (62 years) or in the BREATHE Registry (64 years).^4^ This might explain the cut-off point of 77 years in the survival tree. In addition, there was a predominance of the male sex among patients with HFmrEF and HFrEF.^13^

The infectious causes, septicemia and pneumonia, were listed as having the highest in-hospital specific mortality in the sample. A study^14^ has shown that the cardiovascular prognosis of recent-onset HF improved substantially from 2002 to 2014 (hazard ratio: 0.73; 95% CI: 0.68-0.80) for patients younger and older than 80 years. However, among those older than 80 years of age, the drop in cardiovascular mortality was totally compensated by non-cardiovascular mortality, in which case, the treatment changed the way elderly patients died, as observed in our study.

The presence of dementia syndrome, especially not related to the use of vasopressors on admission, was a factor of worse prognosis evidenced on the survival analysis. A recent study has reported functional decline in 15% of the patients, and, in 80%, that decline occurred prior to admission from decompensated HF and associated with a higher long-term risk for outcome composed by hospitalization and all-cause or HF death, similarly to our findings.^15^

The presence of previous HF in this sample was related to higher mortality, as well as to readmissions due to HF and need for inotropic agents, identified by use of the machine-learning technique. These three variables indicate worse prognosis of patients admitted with decompensated HF and are markers of severity that do not depend on EF. Patients admitted due to HF have a high rate of re-hospitalization in up to 6 months (30% to 40%),^16^ and the risk of death after hospitalization due to HF remains increased from 12 to 18 months from the index event,^17^ being one of the variables used to indicate heart transplantation.^18^ The rates of readmission due to HF in young adults are similar to those of the elderly, suggesting that the re-hospitalization risk is present regardless of age.^19^

Chronic kidney dysfunction and HF often coexist and share several risk factors, such as diabetes, hypertension and hyperlipidemia, which compound the prognosis of decompensated chronic HF.^20^ In addition, the cardiorenal syndrome, characterized by kidney function worsening during hospitalization due to HF or right after discharge, contributes to worsen the prognosis of decompensated HF.^21^ Creatinine level on admission greater than 1.48 mg/dL has been associated with worse prognosis in individuals under the age of 77 years, representing a higher risk for kidney dysfunction, cardiorenal syndrome and need for dialysis.

There are several models to predict mortality from HF, such as the *Get With the Guidelines-Heart Failure* (GWTG-HF)^22^ and the *Meta-Analysis Global Group in Chronic Heart Failure* (MAGGIC),^23^ with unsatisfactory accuracy and without validation for the Brazilian population. Algorithms using deep learning, such as DAHF, improved the ability to predict mortality from HF during hospitalization and after 12 and 36 months from admission; however, they have not been developed for the Brazilian population.^24^ This study’s strength resides in the selection, through Elastic Net and survival trees (machine learning), of patterns of clinical presentation associated with worse in-hospital and late mortality in patients admitted with decompensated HF to a Brazilian cardiac intensive care unit.

One limitation of this study is its single-center nature, in addition to the lack of information on all the medications used prior to admission, such as diuretics. Thus, there is a potential bias of selection inherent in observational studies. There is, in the multiple analyses of the independent variables and mortality, exploratory nature. These characteristics might hinder the external validity of the findings. Regarding internal validity of data, some statistics, such as means and relative risks, are more important. The hypothesis that the EF categorization would be a predictor of in-hospital and late death in this sample was not corroborated by the analysis using machine learning. In this context, death related to decompensated HF seems to represent the sum of aging and progressing organ failures.

## Conclusion

There was no statistical difference in mortality between the groups in the follow-up of 2.94 ± 2.55 years. The survival curve showed difference neither between the HFpEF and HFmrEF groups, nor between the HFpEF and HFrEF groups, but between the HFmrEF and HFrEF groups. Age older than 77 years, HF prior to admission, history of readmission, dementia, creatinine level on admission greater than 1.48 mg/dL, and need for vasopressors were associated with higher late mortality on the survival tree.
